# Delayed diagnosis of pediatric intra-articular epithelioid sarcoma: a case report and literature review

**DOI:** 10.1186/s12887-023-04305-6

**Published:** 2023-09-26

**Authors:** Ranran Zhang, Jia Liu, Lin Liu, Yi Lin, Qiuye Zhang

**Affiliations:** Department of Pediatric Nephrology, Rheumatology and Immunity, The Affiliated Hospital of Qingdao University, Qingdao, China

**Keywords:** Epithelioid sarcoma, Monoarthritis, INI-1, Case report

## Abstract

**Background:**

Epithelioid sarcoma (ES) is a rare form of mesenchymal malignancy that rarely occurs in children. Only seven cases of intra-articular epithelioid sarcoma have been reported in the medical literature.

**Case presentation:**

In this report, we presented the case of a 13-year-old girl with a delayed diagnosis of ES in the left knee. Her initial diagnosis was mistaken for Pigmented Villonodular Synovitis (PVNS) but ruled out later by the first biopsy. However, the lesion rapidly regrew again after arthroscopy, raising suspicions of malignancy. A comprehensive histochemistry examination was conducted again, leading to the diagnosis of INI-1 negative epithelioid sarcoma. Unfortunately, the girl passed away seven months later due to early metastasis of the tumor.

**Conclusion:**

Careful consideration should be given to the differential diagnosis of pediatric patients presenting with monoarthritis. This report highlights the importance of early and accurate diagnosis and underscores the necessity for effective treatments for epithelioid sarcoma. Surgical resection or radical surgery is recommended, while novel treatment strategies targeting EZH2 show promise.

## Background

Epithelioid sarcoma (ES) is a clinically rare malignancy sarcoma first described by Enzinger in 1970 [1]. This mesenchymal malignancy constitutes about 1% of soft-tissue sarcomas and can be subdivided into two subtypes- the distal and proximal types [2–4]. The former, or typical type, primarily affects young adults and adolescents aged from 10 to 40 years old [5, 6]. Typical ES mainly occurs in the extremities, with a predilection for the forearms and hands, while the proximal ES is more easily seen in middle-aged adults and affects axial proximal regions [7, 8]. This malignancy is quite unusual, and intra-articular epithelioid sarcoma is extremely rare, with only a few documented cases in the medical literature [9–14]. Physicians and researchers continue to seek a comprehensive understanding of the causes, mechanisms, and effective treatments for this disease.

In this report, we discussed the case of a 13-year-old girl with a swollen left knee. Initially misdiagnosed as Pigmented Villonodular Synovitis (PVNS) due to unspecific magnetic resonance imaging results, she received arthroscopy. The first pathological tests ruled out PVNS and suggested unspecific synovial membrane inflammation. Unexpectedly, the lesion quickly regrew after the arthroscopy, which raised strong suspicions of malignancy, prompting a second biopsy. Comprehensive pathological and immunohistochemical tests were conducted to confirm the presence of the tumor. Distinct from typical ES patients, this case was quite rare because ES is not typically found in the articular synovial tissue. Such cases have only been infrequently reported over the past decades, with the girl being the first pediatric case. Unfortunately, the girl’s condition progressed quickly, leading to her demise because of tumor malignancy and early metastasis. Her case highlighted the importance of timely and accurate diagnosis and the urgent need for effective treatments for this rare, aggressive cancer form. To better understand this disease, we reviewed relevant literature and summarized the characteristics of intra-articular synovial ES.

## Case presentation

### First hospitalization

A 13-year-old female was firstly hospitalized in September 2021 with a chief complaint of knee joint pain that aggravated after prolonged standing or walking and relieved after rest. The pain had persisted since a traumatic incident five months earlier. Her symptoms worsened with obvious activity limitation ten days ago before she came to our hospital. Physical examination showed a positive McMurray sign and limited range of motion (ROM) of the right knee (60 °-110 °) without other abnormal movements. Magnetic resonance imaging (MRI) indicated a large amount of fluid signal shadow in the right knee joint cavity, suggesting synovitis or PVNS. Synovectomy via arthroscopic surgery was performed for both diagnostic and therapeutic purposes. The patient experienced pain alleviation after the lesion tissues were excised and medivinal drugs (sodium hyaluronate, ropivacaine) were injected into the right knee cavity. Pathological results revealed chronic inflammatory changes in the synovial tissue, characterized by synovial cell proliferation, interstitial collagenization, mucinous degeneration, and focal granulation tissue hyperplasia. PVNS was excluded due to the lack of hemosiderin-laden histiocytes. After the operation, the right knee pain was relieved, and the range of motion improved, so she was discharged.

### Second hospitalization

**Fig. 1:**
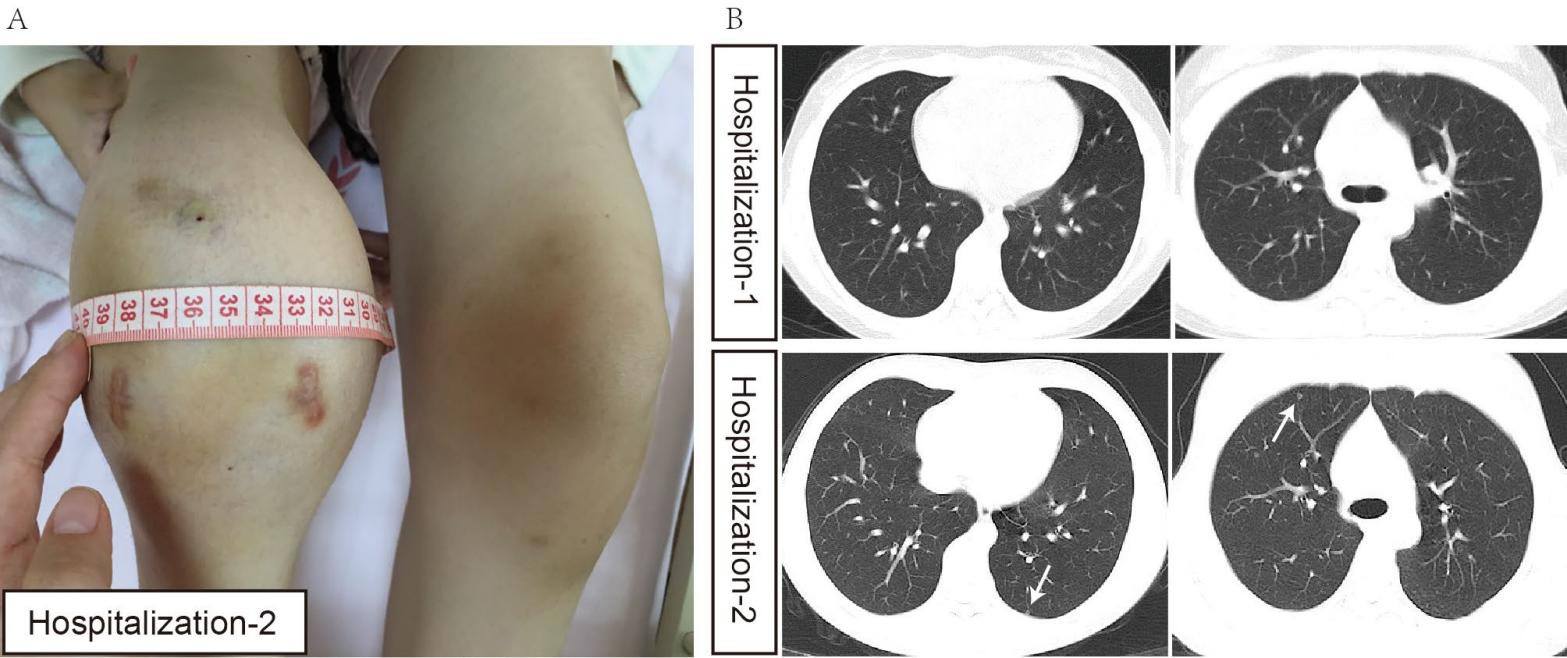
Clinical features of the case. **(A)** Clinical view of the right knee joint, obviously swollen compared to left knee joint. **(B)** Computed Tomography (CT) scan showed multiple small nodules (white arrows) in both lungs in second hospitalization

**Fig. 2:**
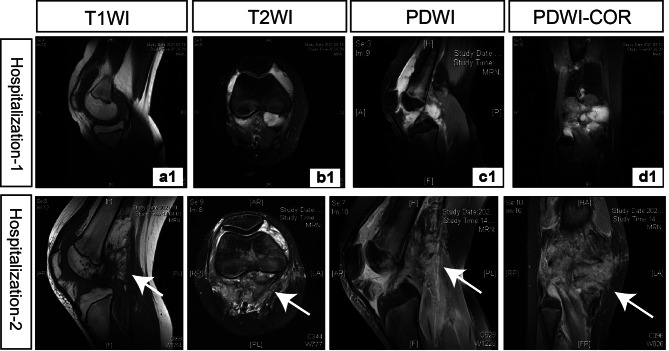
The MRI images of the right knee from different positions. (a1-d1) In the first hospitalization, the MRI images showed a large amount of fluid signal shadow in the right knee joint cavity from different positions, including the lateral sagittal position (a1, T1WI; c1 PDWI), the transverse axial position (b1, T2WI) and the coronary position (d1, PDWI). (a2-d2) During the second hospitalization, the MRI images highlighted patchy high signal shadows (white arrows) in the surrounding soft tissues of the right knee (observed in a2, T1WI; b1, T2WI; c2 PDWI; d2 PDWI-COR). A patchy low T1-weighted signal (a2) and high PD-weighted fat-suppressed signal (c2, d2) were seen in the distal metaphysis of the right femur and proximal metaphysis of the tibia, with unclear borders. Fluid signal shadows and strip-like signal shadows were seen within the right knee joint cavity. Abbreviations: T1WI, T-weighted image; T2WI, T2-weighted image; PDWI, proton density-weighted image; PDWI-COR, coronary proton density-weighted image

Three weeks later, the patient was readmitted due to the recurrence of much more severe pain, accompanied by local swelling, increased skin temperature and limited right knee motion (Fig. 1). Another MRI test revealed extensive thickening of synovial soft tissues around the right knee, with erosion into the lower femoral and upper tibial metaphysis area (Fig. 2). Moreover, fluid signal shadows were observed in the right knee joint cavity.

Laboratory tests showed moderate anemia with a 70 g/L hemoglobin level. Bone marrow film showed hyperplasia of marrow cells and an inverted granulocyte/erythrocyte ratio. Some other routine tests, including erythrocyte sedimentation rate, blood coagulation, liver and kidney function, were normal. Immunologic indicators such as rheumatism indicators, antinuclear antibody, extractable nuclear antigen (ENA) antibody profile, Coombs’ test, and HLA-B27/B7 were all negative. Etiological detection ruled out tuberculosis, Epstein-Barr virus (EBV), and Brucella infections. As the initial pathological result indicated chronic inflammatory changes in the synovial tissue, the findings were regarded as compatible with oligoarticular juvenile idiopathic arthritis. Oral non-steroidal anti-inflammatory drugs (NSAIDs) were adopted, slightly relieving the right knee joint pain. However, the pain worsened two weeks later, accompanied by aggravated swelling and local fever. The corticosteroid treatment was attempted for one week but didn’t work too. Throughout the hospitalization, disease progression was closely monitored via twice joint ultrasounds conducted within a one-week interval. The ultrasounds revealed severely diffuse thickening synovium of the joint along the surgical scar and subcutaneous tissue, with local nodular protrusions. Therefore, a second biopsy guided by ultrasound was performed to confirm the diagnosis.

### Pathologic examination

**Fig. 3:**
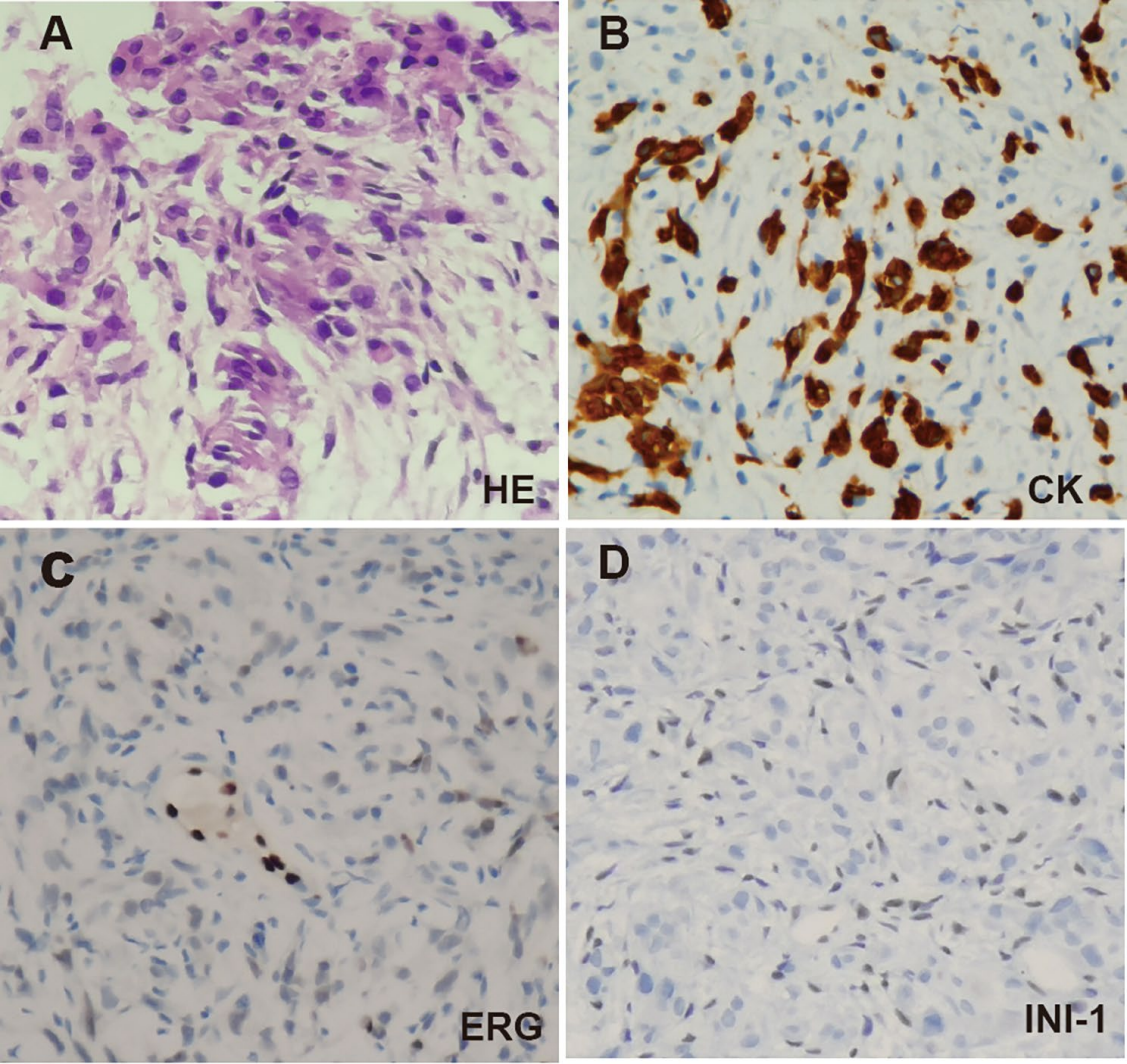
The pathologic findings of the ES patient. **(A)** The biopsy specimen showed epithelial-like cells growing in a streak or patch pattern with vesicular nuclei, prominent nucleoli, atypical cell shape and nuclear division (H&E, 400×). IHC staining showed positive for CK (**B**, 400×) and negative for INI-1(**C**, 400×) and ERG (**D**, 400×)

The synovial membrane biopsy specimen exhibited epithelial-like cells growing in a streak or patch pattern, as revealed in Fig. 3. Some cells were spindle-shaped with eccentrically-located nuclei, frequently showing atypical features and nuclear division. Hyperplasia of interstitial fibrous tissue with mucinous degeneration and focal necrosis was also observed. Immunohistochemistry (IHC) staining showed positive results for epithelial tumor markers (CKpan) and an unspecific marker (SATB2), while staining was negative for CD68, S100, SMA, Desmin, Myogenin, MyoD1, ERG, and TLE1 (Fig. 3). The SMARCB1/INI1 protein, a molecular hallmark of ES, was completely absent, and the proliferating marker Ki67 exhibited a high nuclear staining rate of 30%. To validate the immunohistochemical findings, another fluorescence in situ hybridization detection (FISH) test was performed for *SYT *gene, resulting in a negative result (which meant no SS18 gene-related translocation) that excluded the possibility of synovial sarcoma. In consideration of all pathologic and IHC findings, a diagnosis of INI-1 negative epithelioid sarcoma was indicated.

### Outcome

Afterward, the patient’s parents consulted the pathological findings in the Affiliated Tumor Hospital of Fudan University, where the rechecked immunohistochemistry results (AE1 / AE3 +, INI-1 -, CD34 -) confirmed the diagnosis of INI-1 deficient epithelioid malignant tumor, suggestive of epithelioid sarcoma base on the tumor’s location. Surgery or chemotherapy was recommended; however, she lost the chance of palliative surgical treatment due to the distant metastasis of the lymph nodes, bones, and lungs. One month later, following her initial diagnosis, the girl was transferred to the orthopedic department of Shanghai Sixth People’s Hospital, where the medical team recommended a course of chemotherapy with a prescription of 160 mg albumin paclitaxel and 1.1 g gemcitabine. However, the girl experienced severe adverse effects after the first course of chemotherapy, which prevented her from receiving any further scheduled chemotherapy sessions. Her parents faced a difficult and heart-wrenching choice to go back home and decided to seek palliative treatment in traditional Chinese medicine. The girl’s condition continued to deteriorate steadily, and she passed away seven months after the diagnosis.

## Discussion and conclusions

Typical ES presents as deep slow-growing, painless dermal or subcutaneous solitary nodules or cutaneous ulceration, which mainly affects the tendons or aponeuroses tissues of the extremities, with a predilection for forearms and hands [5, 15]. However, due to its complex histomorphology and slow growth over years, epithelioid sarcoma can affect anyone anywhere and is often misdiagnosed on the first encounter [16, 17]. ES spreads quickly through hematogenous and lymphatic system, and the overall 5-year survival rate is about 32–78% [16]. This paper described the history and clinical features of a 13-year-old girl who was sequentially misdiagnosed as PVNS and juvenile idiopathic arthritis. Unlike other typical cases, the tumor in this girl emerged within her right knee joint, exhibiting an unusually rapid progression and early metastasis that tragically resulted in a rapid demise. Consequently, making an early diagnosis and being acquainted with a relative differential diagnosis is of vital importance. Thus we will also talk about the differential diagnosis of monoarthritis lesions within the scope of this paper.

ES primarily affects the fasciae, subcutaneous tissues, and tendon sheaths of the extremities, displaying multifaceted clinical nature [4, 16]. Some lesions are small, subcutaneous, discrete, and homogeneous, and some lesions are large, deep, infiltrative, and heterogeneous. Accordingly, no distinct MR imaging characteristic would enable its early specific diagnosis [18]. Like other malignancies, the T2-weighted signal characteristics correlate with the proportion of tumor cellularity relative to fibrosis, necrosis, and inflammation [19]. For this reason, the final diagnosis of ES depends on the histopathological examination results. Typically, classic ES appears as multinodular, uniform epithelioid cells with abundant eosinophilic cytoplasm and central necrosis [7]. Proximal ES reveals rhabdoid cells, which are characterized by the presence of large sheets of atypia [7]. According to the former study, the IHC staining of ES demonstrated both epithelial characteristics with positive expression of EMA and low or high molecular weight cytokeratins and CD34 expression, which correlated with interstitial tumors [20]. Loss of SMARCB1 (INI-1) IHC expression is characteristic of both conventional and proximal-type ES and is seen in > 90% of cases [6, 20]. INI-1 was one part of the SWI/SNF chromatin-remodeling complex, encoded by the *SMARCB1* gene, which is located on 22q11.23, involved in coordinating regulation of gene expression programs, and essential in lineage specification and maintenance of stem cell pluripotency [21]. INI-1 is ubiquitously expressed in the nuclei of all normal cells, and the absence of INI-1 can be seen in a variety of tumors, such as malignant rhabdoid tumors, renal medullary carcinoma, and a subset of epithelioid malignant peripheral nerve sheath tumors, myoepithelial carcinomas, and extraskeletal myxoid chondrosarcomas [20]. In ES, INI-1 protein loss may be occurred due to biallelic SMARCB1 or monoallelic deletions with heterogeneous FISH patterns. For our case, IHC staining results prone to define her as INI-1 deficient tumors, and she was finally diagnosed as epithelial sarcoma by further test of LSI SS18 (18q11.2), and a negative result excluded synovial sarcoma.

Given ES’s scarcity, extremely rare evidence was stated about synovial epithelioid sarcoma, especially the intra-articular ones. Up to now, only seven cases have been reported with intra-articular ES, and all were adult patients [9–14]. The patients presented several characteristics (see in Table 1): (1) All patients experienced prolonged nonspecific knee joint pain before seeking medical attention, highly related to trauma or physical activity; (2) Non-specific imaging manifestations led to a high misdiagnosis rate, with two of 7 cases misdiagnosed as PVNS, two as synovitis, and one as osteosarcoma; (3) Pathological and immunohistochemical evidence was required to make a definitive diagnosis; (4) The above-knee amputation(AKA) rate was notably high, with 3 cases applied the AKA operation, two patients undergoing local curative resection, while 2 cases (unable to undergo amputation) opted for chemotherapy or radiation therapy.

**Table 1:** The clinical features and course of reported intra-articular epithelioid sarcoma cases

Study	Age/sex	Symptoms Duration	Imaging	Initial diagnosis Diagnostic approch	Immunohistochemistry	Final diagnosis Treatment	Outcome
Von Hochstetter et al. (1995) (14)	27/F	Right knee Pain and swelling 6 months	Plain X-ray revealed an irregular, flocculent calcification in the infrapatellar fat pad	Osteosarcoma probably arising out of synovial chondromatosis Synovectomy	cytokeratin (+), vimentin (+), CD31 (-)	Radical synovectomy Surgical excision	Developed recurrent soft tissue nodular tumor deposits around the right knee with involvement of joint capsule 4 year later
Hurtado et al. (1998)(12)	35/M	Left knee Pain 10 months	MRI showed prominent synovial proliferation. Bone scan showed soft tissue uptake at left knee	PVNS (hemorrhagic polypoid synovitis with serosanguineous fluid) Arthroscopy	cytokeratin (+), EMA (+), S100 (-), HMB-45 (-)	Above-knee amputation	One lymph node metastatic involvement and no more details stated
Kosemehmetoglu et al. (2011)(13)	19/M	Right knee Pain and swelling 6 years after injury	CT & MRI showed 15 × 13 × 11 cm tumor in knee joint with bone destruction & soft tissue extension	Proximal type ES Not stated	Diffuse cytokeratin (+), variably CD34 (+), complete loss of INI1	Above-knee amputation for ES	Performed well after 1 year without recurrence or metastasis
	60/F	Right knee mass	Radiographic features not stated and not shown, just described as an intra-articular mass	Proximal type ES Not stated	cytokeratin (+), INI-1(-)	Subtotal excision of synocial mass for ES	Bilateral subpleural metastatic nodules at presentation persisted at follow-up 2 years after diagnosis
Chow et al. (2015)(11)	59/F	Right knee Pain and swelling 6 months after falling	MRI showed synovial thickening and lobulated synovial mass	PVNS Open anterior and posterior synovectomy	CD34、EMA、cytokeratin (+), Ki67 (67%+) S100, CD31, desmin & MSA(-) complete loss of INI1	Above-knee amputation for synovial ES	Died of disseminated metastasis 20 months after initial presentation
Martins Rocha T et al. (2018)(9)	22/M	Left knee Pain and swelling 1 year for no reason	MRI revealed minimal joint effusion, a Baker cyst and a chondral fissure of the patella	Unspecific synovitis arthroscopy	Vimentin (+), cytokeratin (+), EMA (+), S100 (-) and complete loss of INI1	Radiotherapy and neoadjuvant chemotherapy (doxorubicin and ifosfamide Above-knee amputation	Not stated
Flikweert et al. (2018)(10)	60/F	Right knee 6 months	MRI revealed a degenerative medial meniscus, popliteal ‘synovial chondromatosis’, and anterior cruciate ligament thickening	Posttraumatic synovitis arthroscopic partial medial meniscectomy, debridement of chondrocalcinosis and synovium in the femoral notch	CD34 (+); AE1 (+); EMA (+)	Palliative care was initiated focusing on pain relief. Due to the rapid clinical deterioration, she received a chordotomy instead.	The patient deceased three weeks after the definitive diagnosis, 5 and 6 months after her first visit to hospital and her family physician, respectively.
Zhang et al. (2023)	13/F	Right knee Pain and swelling 6 months	MRI showed synovial thickening and effusion	Synovitis 1. PVNS? 2. sJIA? Arthroscopy	CKpan, AE1/AE3 (+); CD34, INI-1, CD68, S100, SMA, Desmin, Myogenin, MyoD1, ERG and TLE1 (-)	Due to the metastasis and intolerance of chemotherapy, she mainly adapted palliative care focusing on pain relief.	Died of disseminate metastasis of lymph nodes, lungs and bones 7 months after initial presentation

Note: We created this table based on the previous literature from Chow et al. [11]

Here we presented the first pediatric case of 13 year old, characterized by the onset of chronic monoarticular knee pain. Before being diagnosed as ES, she experienced a series of misdiagnoses. Firstly, due to her long-time engagement in dance practice and exacerbated pain during exertion, her family initially attributed it to overexertion. Approximately six months later, when she initially consulted in our hospital, the first MR imaging only showed synovial fluid accumulation in the right knee joint. Considering the age and clinical manifestations, the doctors mostly suspected PVNS. Timely arthroscopic examination and biopsy were conducted, and the histological findings demonstrated unspecific synovitis without further IHC confirmation. Around one week after arthroscopic surgery, the patient experienced exacerbated pain and noticeable swelling in the left knee joint. A second MRI revealed a significant progression of synovial pathology compared to the previous assessment. Following a multidisciplinary consultation and observing a decrease in hemoglobin levels alongside the MRI findings, the medical team suspected oligo-juvenile idiopathic arthritis (OJIA). However, as anti-inflammatory and analgesic treatments did not work, a second biopsy through ultrasound-guided aspiration was conducted. Whole-scale immunohistochemistry was performed, and the patient was finally diagnosed with epithelioid sarcoma of the synovium in the right knee joint.

It is worth noting that all eight reported cases (including the case in this report)of joint neoplasm occurred in the knee joint, which may suggest that factors such as exercise and occupational strain are one of the inducements of ES. Initially, most patients presented with symptoms such as pain or swelling, persisting for months or even years before being diagnosed as ES, and deteriorated rapidly after the operation or arthroscopic intervention, resulting in a disastrous outcome. Based on the documents, the involvement of a single joint is often attributed to benign factors such as infections, trauma, inflammation, or degeneration [11, 19, 22]. Tumors are considered extremely rare and typically considered as a last resort in the diagnostic process, thus leading to a high rate of misdiagnosis. This is precisely why we initially overlooked the possibility of ES in the girl’s case during her first hospitalization and did not conduct the necessary pathological examinations. It’s noteworthy that invasive procedures are likely to exacerbate the metastasis and progression of bone tumors, while the male sex may also be a factor [11]. For this reason, non-invasive diagnostic clues are even more significant in early diagnosis.

Typically, when monoarticular joint involvement occurs in preadolescence, several clinical conditions should be considered [22], including: (1) infections, such as tuberculosis and bacterial infections, usually display signs of fever, localized tenderness, and elevated inflammatory markers; (2) autoimmune diseases, including juvenile idiopathic arthritis, systemic lupus erythematosus, etc., which often manifest as systemic symptoms; (3) granulomatous lesions, like granuloma annulare, necrotizing granuloma, whose diagnosis relied on the pathological examination; (4) PVNS, a relatively rare benign synovial disease, presents the MR imaging manifestations as a localized nodular or diffuse synovial thickening, with low signal intensity on T1/T2-weighted images; (5) other malignant tumors, such as vascular neoplasm, synovial sarcoma, fibrosarcoma, angiosarcoma, amelanotic melanoma, squamous-cell carcinoma, etc. [7, 22]. In one retrospective research article about pediatric solid intra-articular masses, PVNS mostly encountered solid intra-articular mass lesions in children [22]. That’s why the doctors prompted to suspect PVNS as the primary consideration due to the initial MRI results and the site of onset. OJIA was also a significant concern in the rheumatology department when a single joint exhibited nonspecific inflammation, excluding other potential causes. However, as the symptoms of swelling and pain worsened significantly, it became crucial for us to reevaluate the diagnostic process and imperative to conduct a repeat pathological examination.

Learning from the lessons of this report, rare medical conditions based on common symptoms can be prone to misdiagnosis or oversight. The influencing factors encompass not only the clinical features and the auxiliary examination results but also the level of awareness among specialized medical professionals, patient cooperation, and economic factors. Accurate and differential diagnoses depend on thorough evidence, such as systematic medical history collection, physical examinations, characteristic radiological imaging features, and comprehensive or specific molecular pathological evidence. However, in practical clinical settings, physicians often tend to make initial judgments based on their clinical experience. At the same time, the patients refused to accept comprehensive examinations for various factors, leading to diagnostic errors.

ES is prone to develop occult metastasis in the early stage of the disease, resulting in a poor prognosis and a very high disability or mortality rate. The recurrence rate is also notably elevated, reaching up to 50%, frequently manifesting within a two-year timeframe [16, 18]. Metastases primarily affect the pulmonary system, as well as the lymph nodes, brain, bone, and scalp [5, 16]. To effectively address ES, medical practitioners typically advocate for extensive surgical resection or radical surgery whenever feasible, aiming to excise a maximal extent of malignant tissue and curtail further disease dissemination. Patients grappling with surgically unresectable local recurrence or metastatic disease often receive conventional cytotoxic chemotherapy or tyrosine kinase inhibitors as standard treatment options [6]. However, the clinical benefits from these interventions may be modest, constantly proving insufficient to enhance the patient’s overall condition significantly. Recently, new efforts have been put into novel therapeutic avenues, specifically targeting EZH2 [20, 23, 24]. Noteworthy among them was tazemetostat, which demonstrated enhanced tolerability in ES patients with a deficiency in INI1/SMARCB1 [23, 24]. In addition, a more profound exploration into the genomic or epigenomic alterations, undertaken through fundamental research endeavors, holds the potential to unlock broader vistas of opportunity.

## Data Availability

Not applicable.

## Abbreviations

ES: Epithelioid sarcoma
PVNS: Pigmented Villonodular Synovitis
ROM: Range of motion
MRI: Magnetic resonance imaging
CT: Computed Tomography
ENA: Extractable nuclear antigen
NSAIDs: Non-steroidal anti-inflammatory drugs
IHC: Immunohistochemistry
FISH: Fluorescence in situ hybridization detection
O-JIA: Oligo-juvenile idiopathic arthritis
